# Unexpected cause of lower limb edema

**DOI:** 10.11604/pamj.2024.47.210.43390

**Published:** 2024-04-24

**Authors:** Kaoutar Khabbache, Abdallah Oulmaati

**Affiliations:** 1 Faculty of Medicine and Pharmacy of Tangier, Abdelmalek Essaâdi University, Tangier, Morocco,; 2 Department of Pediatrics, University Hospital Mohammed VI Tangier, Tangier, Morocco

**Keywords:** Fabry disease, angiokeratoma, lower limb edema, lymphedema

## Image in medicine

A 16-year-old male patient, with no notable personal medical history, was consulted for edema in the lower limbs. The familial medical history reveals a 25-year-old maternal uncle undergoing treatment for renal insufficiency at the hemodialysis stage. The onset of symptoms traces back to the age of 13 when the child experienced episodes of intermittent painful edema in the lower limbs. This prompted the patient to consult several doctors and undergo various assessments; inflammatory, renal, hepatic, and cardiac functions were all found to be normal. The clinical examination revealed unilateral edema in the left lower limb extending to the ankles. Additionally, multiple millimeter-sized erythematous maculo papular lesions were observed on his trunk, back, and umbilicus, corresponding to diffuse angiokeratomas, while the rest of the clinical examination was unremarkable. Fabry disease, a lysosomal disorder, was suspected due to the history of renal disease in a young relative and the presence of lymphedema and angiokeratoma. Confirmation of Fabry disease was achieved through enzymatic assay, indicating a high value of Lyso-GL-3 level (90.5 ng/ml, with a cut-off value of 0.0-3.5), and further supported by a molecular study revealing the presence of a hemizygous mutation c.602C>T (p. (Ser201Phe)). Cardiac ultrasound and ophthalmic assessment yielded normal results, and renal function showed no abnormalities with negative proteinuria. The patient was proposed for enzyme replacement therapy.

**Figure 1:**
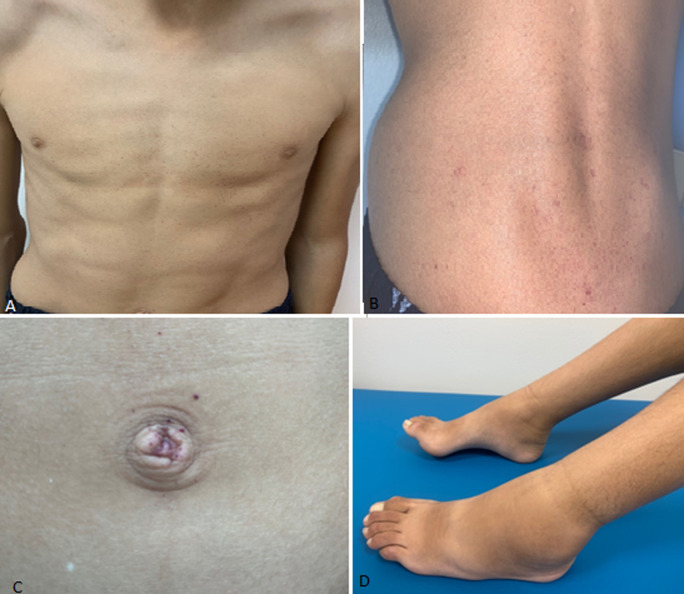
A) diffuse angiokeratoma on the thorax and abdomen; B) angiokeratoma on the back; C) angiokeratoma on the umbilicus; D) edema in the left lower limb extending to the ankle

